# Operon-based approach for the inference of rRNA and tRNA evolutionary histories in bacteria

**DOI:** 10.1186/s12864-020-6612-2

**Published:** 2020-04-16

**Authors:** Tomasz Pawliszak, Meghan Chua, Carson K. Leung, Olivier Tremblay-Savard

**Affiliations:** Department of Computer Science, University of Manitoba, Winnipeg, Canada

**Keywords:** Operons, rRNA and tRNA genes, Phylogeny, Evolutionary histories, Ancestral gene orders, Global alignments

## Abstract

**Background:**

In bacterial genomes, rRNA and tRNA genes are often organized into operons, i.e. segments of closely located genes that share a single promoter and are transcribed as a single unit. Analyzing how these genes and operons evolve can help us understand what are the most common evolutionary events affecting them and give us a better picture of ancestral codon usage and protein synthesis.

**Results:**

We introduce BOPAL, a new approach for the inference of evolutionary histories of rRNA and tRNA genes in bacteria, which is based on the identification of orthologous operons. Since operons can move around in the genome but are rarely transformed (e.g. rarely broken into different parts), this approach allows for a better inference of orthologous genes in genomes that have been affected by many rearrangements, which in turn helps with the inference of more realistic evolutionary scenarios and ancestors.

**Conclusions:**

From our comparisons of BOPAL with other gene order alignment programs using simulated data, we have found that BOPAL infers evolutionary events and ancestral gene orders more accurately than other methods based on alignments. An analysis of 12 *Bacillus* genomes also showed that BOPAL performs just as well as other programs at building ancestral histories in a minimal amount of events.

## Background

With all the advancements in sequencing and culturing methods, coupled with burgeoning interests in gut microbiomes [1, 2] and transmission risks of pathogenic microbes [3, 4], bacterial genomes are now being sequenced at a very fast pace. This wealth of genetic information provides a great opportunity to study bacterial genome evolution, compare evolutionary rates between different genera, and study the prevalence, frequencies and average size of different evolutionary events.

An interesting aspect of bacterial genomes is the presence of operons [5] (operons have been identified more recently in eukaryotes [6], but they seem to be more prevalent in prokaryotes). An operon is basically a cluster of closely located genes (also called polycistronic genes) that share a single promoter and are transcribed simultaneously into a single polycistronic messenger RNA (mRNA). These genes can then be translated together, or separately when spliced into separate mRNAs (differential expression of polycistronic genes has also been observed [7]). Some of the most studied and well-defined operons in bacteria are ribosomal RNA (rRNA) and transfer RNA (tRNA) operons [8, 9]. There are several reasons for the interest in these operons, since those genes are fundamental for protein synthesis, and their numbers and organization can be used to better understand codon usage [10]. Comparing the rRNA and tRNA gene contents and organization in different species and inferring evolutionary scenarios allows to make predictions about core sets of tRNA genes, ancestral protein synthesis, ancestral codon usage and evolutionary rates.

In 2012, a new method was developed to infer evolutionary histories of rRNA and tRNA genes that was based on a gene order alignment approach and considered duplication and loss events [11]. The alignment of the gene orders was used to identify orthology relationships between the genes (since there are multiple copies of each type of rRNA and tRNA genes), instead of using traditional methods for identifying gene orthologies from sequence information. The rationale for using this approach is that tRNA genes especially are very short (maximum length is around 90 nucleotides), and the sequences are highly conserved [12]. As a consequence, there is simply not enough signal in the sequences themselves to identify orthology relationships. This alignment problem was then shown to be NP-hard for the duplication and losses model of evolution [13–15]. The exact algorithm proposed in [11], based on integer linear programming (ILP), was designed to solve the 2-Small Phylogeny Problem (2-SPP), which is to find a common ancestor *A* of two gene orders *X* and *Y* that minimizes the number of events on each of the two branches. Andreotti et al. [15] then proposed a faster and more efficient linear programming algorithm for the duplication-loss model, and generalized it to the median of three genomes setting. More recently, OrthoAlign [16] and multiOrthoAlign [17] were developed to generalize the evolutionary model to account for rearrangements (inversions and transpositions) in addition to duplications and losses. The idea there was to use dynamic programming to align the rRNA and tRNA gene orders and identify orthologs, and then explain the mismatches and gaps in the alignment by inferring rearrangement events (inversions and transpositions) and content-modifying events (duplications and losses). While OrthoAlign was designed for pairwise comparisons between gene orders (2-SPP), multiOrthoAlign was created to compare a full set of gene orders related through a phylogenetic tree by taking initial ancestral assignments (inferred by OrthoAlign or another method) and improving them using a heuristic for the median of three problem.

The gene order alignment approach used in the methods described previously is well adapted to the study of bacterial genera which have a relatively low amount of divergence between the genomes. However, just like with traditional sequence alignment, when the gene orders being compared are not very well conserved, it quickly becomes difficult to correctly identify matches (which correspond to orthologous genes in this case). Moreover, existing methods do not consider the physical proximity of the genes in their inference of events, which might lead to evolutionary histories that are not necessarily realistic — inferring events on blocks of genes that are contiguous in terms of gene order but not necessarily close to each other on the chromosome for example.

In this paper, we propose BOPAL (Bacterial OPeron ALigner), a new approach that is designed to consider the organization of genes into operons and to be more flexible to the relocation of operons into different regions of the genome because of rearrangements. Instead of trying to find orthologous genes, which might not be located in the same region in both gene orders being compared, our method is based on identifying orthologous operons of rRNA and tRNA genes. Indeed, these operons tend to be more conserved in general, since rearrangement events mostly change their location inside the genome, but rarely modify their composition (e.g. a rearrangement event is unlikely to split an operon into two parts) [16]. Considering operons also allows our method to infer more realistic events by not considering events that would affect blocks of genes that are not part of the same operon, so technically not close to each other on the chromosome. Our heuristic, which considers duplications, deletions, inversions, transpositions and substitutions, can be used for pairwise comparisons (2-SPP), or it can be used to reconstruct the complete evolutionary history and ancestors of a set of gene orders on a phylogeny by solving instances of the 2-SPP in a post-order traversal of the tree.

We validate our new approach on simulated datasets (cherries and cherries with a neighbor), and we also test it on the same *Bacillus* dataset of 12 genomes used in [15, 17]. Our results show that with our simulated data, BOPAL has the ability to infer events and ancestral gene orders with higher accuracy than other gene alignment algorithms. Similarly, on a biological dataset, BOPAL has performed equally as well as multiOrthoAlign [17] and DupLoCut [15] at generating the ancestral tree in a minimal amount of events.

## Methods

### Evolutionary model

Our evolutionary model is based on the results and observations of previous studies on bacterial genome, operon and tRNA gene evolution, as described below.

In bacterial genomes, genes tend to be located mostly on the leading strand of DNA (pointing away from the origin of replication or in other words pointing towards the terminus of replication) [18], so as to avoid potential head-on collisions between the RNA and DNA polymerases [19]. As observed in a previous study of the *Bacillus* genus [16], inversions are mostly occurring around one of the *axes of replication* (origin or terminus) because this causes the genes to stay on the leading strand.

It was observed in [16] that duplications can either insert the copied genes inside or outside other operons, thus extending pre-existing operons or creating new ones. Also, rearrangements (inversions and transpositions) do not seem to break operons into separate parts. In the study of 50 *Bacillus* genomes, the inferred rearrangements always affected entire operons and not just a part of them [16]. Although these constraints on rearrangements were observed in a study of the *Bacillus* genus specifically, we assume that they can be generalized to other bacteria, since a rearrangement affecting only part of an operon would most likely leave one part of it without a promoter.

Multiple sites in tRNA sequences, extending beyond the anticodon region, are responsible for their recognition by the aminoacyl-tRNA synthetases, which charge the tRNA molecules with the appropriate amino acid [20]. Mutations in these identity elements can sometimes change the identity class of tRNA genes [21, 22], which can be viewed as a substitution to a different tRNA gene.

Based on these observations, our evolutionary model aims to represent *realistic histories*. We define a *realistic history* as an evolutionary history (series of events transforming a genome into another) that considers: (1) the organization of genomes into operons, (2) that rearrangements do not split operons into separate parts, (3) events that move or copy genes across an axis of replication (origin or terminus) reverse the genes, and (4) block (or segmental) duplication/deletion events can only affect genes that are closely located (part of the same operon).

More specifically, our evolutionary model considers the following events:

- A *duplication* copies either a singleton, a gene or a segment of genes inside an operon, or a full operon to another position in the genome. If the duplicated gene(s) are copied to the other side of an axis of replication, an *inversed duplication* occurs, which involves reversing the order and changing the signs (representing transcriptional orientation/strand) of the genes in the duplicated segment.
- A *deletion* (or a loss) removes either a singleton, a gene or a segment of genes inside an operon, or a full operon from the genome.
- An *inversion* (or reversal) reverses the order and changes the sign of the genes affected. Inversion events can only affect singletons or entire operons (not breaking an operon into separate parts), and must occur around an axis of replication, i.e. the segment that is reversed must be immediately next to either the origin or terminus of replication. These constraints are based on the prevalence of these types of inversions as described in [16].
- A *transposition* moves either singletons or entire operons to a different place in the genome (for the same reasons described above for inversions). Similarly to duplications, transpositions that move genes to the other side of an axis of replication will be *reversed transpositions*, also reversing the order and changing the signs of the transposed segment.
- A *substitution* is an event that modifies the anticodon of a tRNA gene and/or reassigns a tRNA gene to another identity class.

### Problem statement

#### Input

The algorithm we propose takes as input a phylogeny representing a bacterial genus, and annotated rRNA and tRNA gene orders, i.e. circular unichromosomal gene orders in which the locations of the origin and terminus of replication, the operons, the anticodons (in the case of tRNA genes), and the signs of the genes have been identified. The rRNA genes and tRNA genes are either part of an operon (polycistronic) or not (monocistronic), in which case we refer to them as *singletons* in this paper. Each gene order for each extant genome studied is associated to a leaf node. For conciseness, in this paper we will not make a distinction between a node and its associated gene order.

#### Problem

The problem is to infer a parsimonious realistic history for the annotated gene orders with duplicates, considering the evolutionary model described above, and ancestral gene orders (corresponding to internal nodes) on the full input phylogeny.

### Annotation of the gene orders

#### Location of the origin and terminus of replication

To annotate the gene orders with the locations of the origin and terminus of replication, we use the SeqUtils module from the Biopython package [23]. The SeqUtils module allows us to calculate the GC skews using a sliding window in the full genome sequences, and identify the minimum and maximum values of GC skews. The extrema of the GC skew function are known to be correlated with the loci of the origin and terminus of replication [24].

#### Location of the operons

Several methodologies have been proposed to find operons in microbial genomes, which are based on several different genomic features like intergenic distances [25], metabolic pathways [26], expression profiles [27], phylogenetic information [28], etc. Since rRNA and tRNA operons do not contain any other types of genes in the biological dataset presented below, we used a simple rule for determining operons: a maximum intergenic size of 200 bp is allowed between each consecutive rRNA or tRNA gene to consider them part of the same operon. Note that more sophisticated approaches, and/or databases of annotated bacterial operons would be necessary if one were to consider all types of operons in the genomes. An even more precise approach would be to consider experimentally identified transcriptional units, such as those integrated into the DOOR 2.0 database of prokaryotic operons [29] (unfortunately, the DOOR 2.0 database was inaccessible at the time of writing).

### Algorithm

**Fig. 1:**
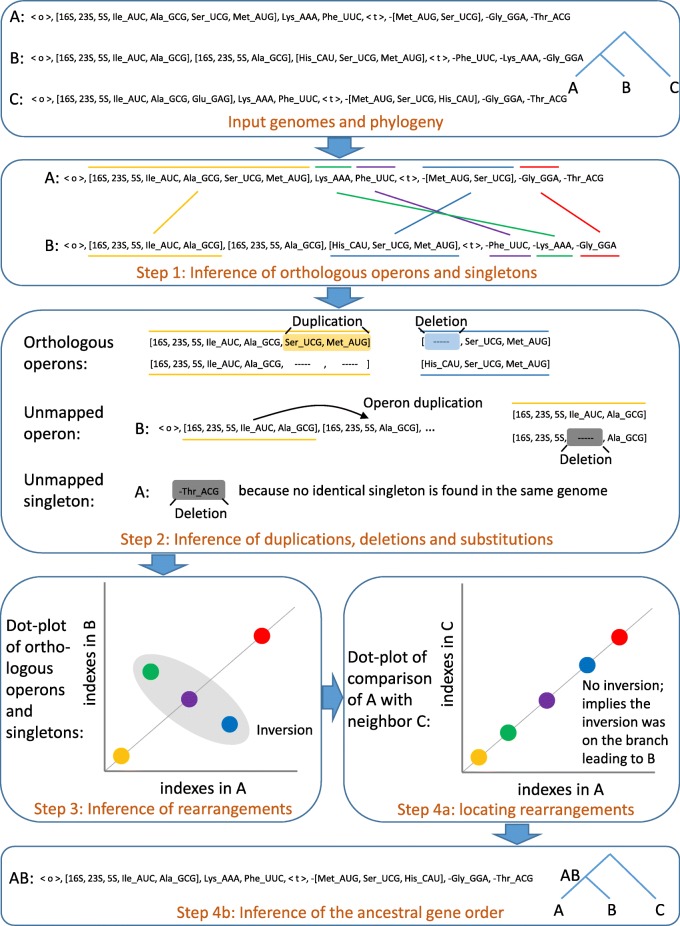
Flowchart describing the 4 main steps of BOPAL on a cherry (*A*,*B*) with a neighboring genome *C*. Operons are enclosed in square brackets, whereas singletons are not, and <*o*> and <*t*> represent the origin and terminus of replication respectively

The proposed approach traverses the whole input phylogeny in post-order, and compares two siblings (left and right child of an internal node, also called *cherry*) at a time to produce an evolutionary scenario and an ancestral gene order for the internal node. Once the ancestral gene order is produced, the post-order traversal continues to produce the next ancestral genomes and so on until the full evolutionary history (on all branches of the phylogeny) has been inferred. Below is a description of the four steps of the algorithm for each comparison of two child nodes (each instance of the 2-SPP), when a neighboring species is available (also see Fig. 1 for a flowchart describing the steps on an example).

#### Step 1: inference of orthologous operons and singletons

We first use all-vs-all pairwise global alignments between the operons of the two genomes compared to identify orthologous operons. This is one of the major differences between our approach and the previous ones presented in [11, 16, 17]: instead of aligning the full gene orders to identify orthologous genes, we align only the operons, which tend to be more conserved. Moreover, the global alignments are not used to label events at this time, but only to find similar operons, which allows us to use a simpler scoring mechanism. Once pairs of orthologous operons have been identified, the matched genes contained in the paired operons are considered to be orthologous.

Let *M* be the dynamic programming table for the global alignment of operons *X* and *Y*, and *M*[*i*−1,*j*−1] be the optimal score of aligning the prefix of *X* ending at position *i*−1 and the prefix of *Y* ending at position *j*−1, the score *M*[*i*,*j*] can be calculated using the following recursive function:

1 $$ M[i, j]= Max \left\{\begin{array}{ll} M[i-1, j-1] + 1, & \text{full match}\\ M[i-1, j-1] + 0.5, & \text{partial match}\\ M[i-1, j-1] - 1, & \text{mismatch}\\ M[i, j-1] - 1, & \text{gap in } {X}\\ M[i-1, j] - 1, & \text{gap in } {Y} \end{array}\right.  $$

where a full match is when both the gene and the anticodon match, a partial match is when the gene matches but with a different anticodon and a mismatch is when both the gene identity class and (necessarily) the anticodon don’t match. This notion of partial match only applies to tRNA genes and not rRNA genes, which are not annotated with anticodons. Note that many different scoring schemes could be used here, as long as the score for a match is greater than the score of a partial match, which itself should be greater than the score of mismatches and gaps. The main assumption for setting the score of a partial match in between the one of a full match and the one of a mismatch is that more mutations (not just in the anticodon) would be necessary to completely change the identity class of a tRNA gene, as opposed to a change in the anticodon that preserves the identity class. We ended up using this specific scoring system because it performed well in practice.

After completing all the comparisons, we discard all pairs that have an alignment score <0. We then label pairs of operons from the two genomes as orthologous starting from the highest alignment scores to the lowest. In case of ties (e.g. an operon from genome *X* aligns with two operons of genome *Y* with the same score), we select the pair of operons that is closest in terms of their respective indexes in the genomes.

As for singletons between the two genomes, we simply label them as orthologous if they are identical (same identity class and same anticodon, in the case of tRNA genes). When there are mutliple choices, we choose the pairs that are located in the same (or most similar) position in the genome based on their respective indexes.

#### Step 2: inference of duplications, deletions and substitutions

During this step, we first infer duplications, losses and substitutions within the orthologous operons, based on the alignments that were made in *Step 1*. Mismatches or partial matches simply correspond to substitutions. Gaps in the alignment can be labeled either as duplications in one genome, or deletions in the other genome. We follow a simple rule for determining if a gap is a duplication or a loss:

- if the gap has a size ≥2 and there exists an identical sequence of genes somewhere else in the same genome, we label it as a duplication;
- otherwise, we arbitrarily label the gap as a deletion.

This simple rule is prone to produce errors, especially for gaps of size one which are always considered to be deletions. The problem with gaps of size 1 is that, since there are almost always multiple copies of each rRNA and tRNA genes in each genome, we could almost always either infer a duplication (recall that to infer a duplication, we must find the same gene — same identity class and anticodon — somewhere else in the genome) or a loss. To alleviate this problem, we allow our algorithm to correct itself by changing deletions into duplications during the next comparison with the neighboring genome, i.e. when we compare the produced ancestor with another sibling (see *Step 4* below for more details).

Once all the orthologous operon pairs have been resolved, we deal with the operons that have not been mapped to an orthologous one in the other genome. We must then infer if these “leftover” operons are the product of a whole operon duplication in one genome (thus being paralogous operons), or a whole operon deletion in the other genome. For each of them, we perform a global alignment with all the other operons within the same genome to find the strongest match with a score ≥0. If it exists, we label the whole operon as being duplicated and then we infer duplications, losses and substitutions to explain the gaps and mismatches/partial matches in the alignment in the same manner described above. This is another strength of our approach, because it allows us to infer *overlapping*, or *non-visible* events, i.e. consecutive events on the same genes that do not directly appear on an alignment of the two genomes. This is another improvement over the previous algorithms, which were designed to consider only visible events [11, 16, 17]. If no match within the same genome is found with a score ≥0, we simply infer that the non-mapped operon was deleted in the other genome. We proceed in the same manner for the non-mapped singletons, except that the alignment is not required: we simply infer them as duplicated if there is an identical singleton in the same genome, and deleted otherwise.

#### Step 3: inference of rearrangements

Another advantage of our approach is that we infer rearrangements independently of duplications, deletions and substitutions, which once again permits the inference of overlapping events, in the sense that a gene affected by a duplication, deletion or substitution can also be affected by a rearrangement.

In this step, we produce a dot-plot representing all the orthologous operons and singletons paired in *Step 1* (each axis represents a genome and there is one dot for each pair of orthologs; see Fig. 1 for an example). We use this dot-plot to identify conserved segments, inversed segments and transposed segments. Just like in any dot-plot, conserved segments are series of dots that are located on the main diagonal. Inversed segments can be identified on the dot-plot as a series of dots that cross the main diagonal in the opposite orientation. The other dots or series of dots which are not found on the main diagonal and not inversed are simply identified as transposed segments (either *forward* transposed or *reversed* transposed, depending on their orientation).

#### Step 4: inference of the ancestral gene order

One important detail about inversions and transpositions, as described in [16], is that they can be applied to any of the two sequences. There is simply not enough information in a pairwise comparison that can allow us to discriminate between the two equally probable scenarios. To identify the genome in which the event occured, we use the same strategy proposed in [16], where we use one of the two sibling genomes *X* and compare it with another neighboring genome *N*. *N* is simply the first resolved genome (either a leaf or a previously built ancestor) encountered in the subtree that is the sibling of the cherry’s parent. If the same segment is found to be inversed (respectively transposed) again in that other comparison, then we know that the event occurred on the branch leading to *X*. Otherwise, if the segment is not inversed (respectively transposed) again, then we know that the event occurred on the branch leading to the other sibling *Y* (see Fig. 1 for an example).

Once all the events have been inferred on the correct branches (leading either to genome *X* or *Y*), the ancestral gene order can trivially be produced simply by “undoing” the events (e.g. a deleted gene will be placed back into the ancestor, etc.). Once the ancestor is produced, the next comparison can be made following a post-order traversal of the phylogeny. Similarly to how we deal with rearrangement events, we use the next comparison with a neighboring genome to potentially correct for errors in inferred deletions. We keep track of all the genes that were added back into the ancestor because of a deletion event, and if they cause a gap in an alignment (during *Step 1*), we replace the previously inferred deletion event by a duplication event and modify the ancestor accordingly.

### Complexity

For each comparison of two child nodes (each cherry), suppose for simplicity that both gene orders contain *n* genes, distributed among *c* operons. On average, an operon will contain about *n*/*c* genes. *Step 1* of the algorithm requires a global alignment of all pairs of operons between the two genomes: there are *c*^2^ such pairs, and each alignment can be done in *O*((*n*/*c*)^2^), which results in *O*(*n*^2^) time for *Step 1*. In *Step 2*, labeling the gaps in the selected alignments (representing orthologous operons) requires scanning the genome for potential sources of duplications: each scan takes *O*(*n*) and there is a maximum of *O*(*n*) gaps in total (because selected alignments must have a score ≥0), which results in *O*(*n*^2^) time. The other part of *Step 2* that identifies whole operon duplications takes *O*(*n*^2^), similarly to *Step 1*, for all the pairwise global alignment of operons within the same genome. Finally, *Step 3* can be done in linear time, and *Step 4* is similar to *Step 1* but with a neighboring genome, so it takes *O*(*n*^2^) as well. This leads to a worst-case complexity of *O*(*n*^2^) for each cherry.

### Potential strategy for dealing with horizontal gene transfer

Although we did not consider horizontal gene transfer (HGT) in our evolutionary model, we propose a potential strategy to infer these events. Assuming that an HGT event could copy an operon from an unrelated genome, which is not necessarily present in the considered phylogeny, this operon would not be mapped to an ortholog in the sibling genome in *Step 1*. In *Step 2*, this operon would probably not be mapped to another operon in the same genome either, which would not allow the algorithm to infer a duplication of the operon. Currently, this would result in the method labeling this operon as lost in the sibling genome, and it would be placed back into the ancestor. However, we could then compare this operon with a neighboring genome *N*, to see if it actually matches. If it matches well with an operon in *N*, then we keep it as lost, otherwise, the algorithm could label it as being the result of an HGT, and similarly to a duplication, the operon would not be added to the ancestor.

## Results and discussion

We implemented our algorithm in Python 2.7 and named it BOPAL — Bacterial OPeron ALigner. We then evaluated it on simulated and biological datasets.

### Evaluation on simulated datasets

We developed a simulated data generator that takes as input a tree topology of *L* leaves, an ancestral genome size denoted by a number of genes *n*, and the number of events to be generated on each branch of the tree *E*. The generator creates a random ancestral gene order (note that we do not simulate sequences, since our approach does not use sequence information, other than the tRNA anticodons), annotated with operons and anticodons, at the root of the phylogeny and randomly simulates evolution of each branch according to the selected parameters. We use a geometric distribution, with a parameter that we named *p*_*op*_, to sample the size of the operons and then we populate them with genes. Singletons are randomly added to genomes using a probability *prob*_*s*_, and the probability of adding an operon instead is 1−*prob*_*s*_. During the simulated evolution, when an event is chosen to be performed on a branch, a random starting point is selected and its size (number of genes or operons affected) is also sampled from a geometric distribution (we named the parameter of this geometric distribution *p*_*event*_). In accordance with the evolutionary model described earlier, the generator will not simulate rearrangements that break operons into separate parts, simulate inversions that are not occurring around an axis of replication, etc.

#### Accuracy on cherries with neighbor

We tested how our new approach compares with the 2-SPP algorithm of [11] (hereafter referred to as DupLoss) and OrthoAlign [16] on cherries, i.e. two sibling leaves that share the same parental node. We also added to our simulations a third neighboring genome to test how OrthoAlign and BOPAL perform with the additional information coming from the neighbor. Note that we did not test the DupLoCut algorithm because the output only reports the total number of events, which would not allow us to analyse all the types of accuracy that we consider below. Also, we were not able to perform tests with multiOrthoAlign because no implementation was available online at the time of writing.

For this test we used a triplet phylogeny (*L*=3 leaves), a constant ancestral genome size *n*=120,*p*_*op*_=0.125 (producing an average operon size of 8.2), *prob*_*s*_=0.35 (resulting in an average number of singletons and operons of 7.8 and 13.7 respectively), and *p*_*event*_=0.7. These probabilities and parameters were chosen to represent as closely as possible the biological dataset studied below (see Table 1 for more information on the biological dataset). As for the simulated events, we used one inversion randomly applied to one of the branches of the cherry, and *x* times a duplication, a deletion, a transposition and a substitution on each branch (so the total number of events per branch are multiples of 4, excluding the single inversion). Note that we simulated only one inversion because our model considers inversions around an axis of replication only, and multiple consecutive inversions tend to cancel each other out. Based on the previous analysis of 50 *Bacillus* genomes [16], inversions do not seem to occur very frequently (only 23 inversions were inferred in total, for an average of 0.232 inversions per branch), which makes the simulation of 1 inversion per cherry reasonable. All the results presented below are averaged over 100 replicates.

**Table 1:** Description of the 12 *Bacillus* genomes studied, their NCBI accession number and information about the annotated rRNA/tRNA singletons (sing.) and operons (op.)

Genome name	Accession #	# of sing.	# of op.	Avg. op. size	% of genes
*Bacillus cereus* ATCC 10987	NC_003909	6	15	8.47	2.46
*Bacillus cereus* E33L	NC_006274	5	16	8.13	2.30
*Bacillus cereus* ATCC 14579	NC_004722	7	15	9.33	2.69
*Bacillus thuringiensis* BMB171	NC_014171	5	17	8.29	2.58
*Bacillus thuringiensis* serovar kurstaki str. HD73	NC_020238	6	15	8.93	2.45
*Bacillus thuringiensis* serovar konkukian str. 97-27	NC_005957	9	14	9.79	2.77
*Bacillus subtilis* subsp. spizizenii str. W23	NC_014479	9	11	8.36	2.57
*Bacillus subtilis* subsp. spizizenii TU-B-10	NC_016047	9	13	8.69	2.99
*Bacillus subtilis* subsp. subtilis str. 168	NC_000964	9	11	9.73	2.56
*Bacillus amyloliquefaciens* FZB42	NC_009725	9	15	7.20	3.17
*Bacillus amyloliquefaciens* subsp. plantarum CAU B946	NC_016784	8	15	7.80	3.30
*Bacillus amyloliquefaciens* DSM 7	NC_014551	9	16	7.19	3.20

The “% of genes” column represents the proportion of all tRNA and rRNA genes over the total number of coding genes in the genome

**Fig. 2:**
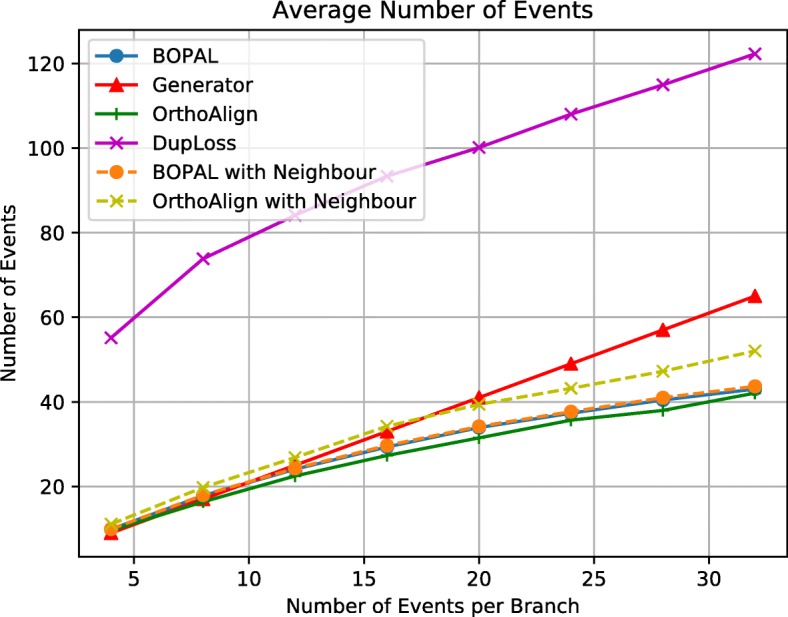
Total number of events inferred, for multiples of 4 events per branch and one inversion on one of the branches leading to the cherry

To measure the accuracy of the different approaches, we first compared the total number of events inferred by the three different methods with the total number of events that were simulated by the data generator (see Fig. 2). Unsurprisingly, DupLoss, which does not consider rearrangements, has to infer a lot more events to explain these evolutionary scenarios. All the other methods tend to underestimate the number of events when more events are generated, which is expected since the traces of some events can disappear after successive events, and some shortcuts can be found in the evolutionary scenarios. The use of a neighbor with BOPAL does not make much of a difference in the total number of events inferred, since the neighbor is used only to place rearrangements on the correct branch and potentially modify a deletion of size 1 into a duplication of size 1. In OrthoAlign however, using a neighbor increases the number of events, probably when it modifies deletions of a block of genes for more smaller duplications.

We also measured how accurate the ancestral gene orders produced were. To do this, we used DupLoss [11] to align the inferred gene order with the simulated one and counted the gaps in this alignment (DupLoss [11] does not allow mismatches and only produces matches and gaps). Matches in this comparison of ancestral gene orders were counted as true positives (TP), gaps in the inferred ancestor, which correspond to missing genes, were counted as false negatives (FN), and finally gaps in the simulated ancestor, which correspond to extra genes, were counted as false positives (FP). These allowed us to calculate recall and precision:

2 $$ recall = \frac{TP}{TP + FN}  $$

3 $$ prec. = \frac{TP}{TP + FP}  $$

We then combined recall and precision into one measure by calculating their harmonic mean, which is traditionally called the F-measure:

4 $$ F = 2 * \frac{recall * prec.}{recall + prec.}  $$

**Fig. 3:**
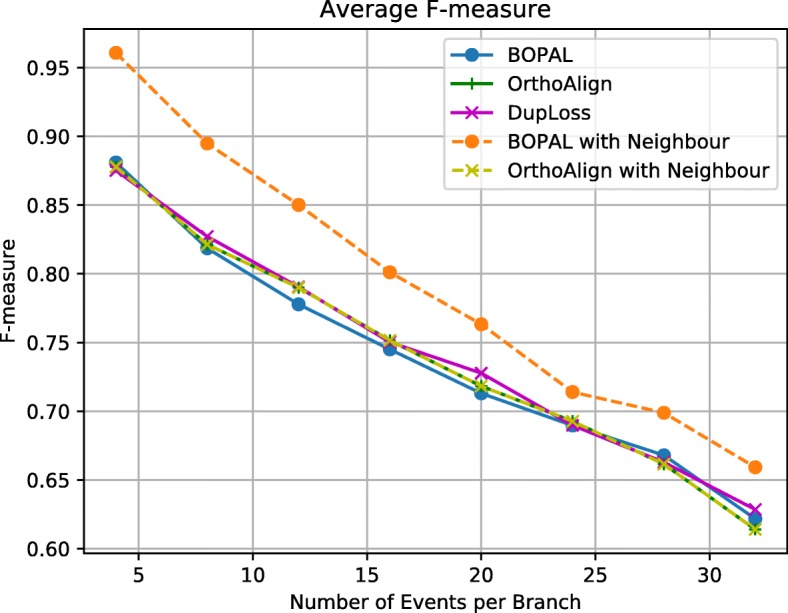
F-measure of the reconstructed ancestral gene orders

Results on the F-measure for the inferred ancestors are presented in Fig. 3. In general, all methods perform similarly, except BOPAL with the neighbor which infers considerably more accurate ancestors. BOPAL without the help of the neighbor seems to perform the worst, however, this was expected, since BOPAL does make some arbitrary choices between deletions and duplications when there is no neighbor, and might infer rearrangements on the wrong branches. Interestingly, having a neighbor does not seem to improve the ancestral prediction of OrthoAlign. As for DupLoss, it performs similarly to OrthoAlign for the F-measure, but it is still reasonably accurate in its inference of the ancestral gene order, even if it has to use a lot more events.

Finally, we measured the accuracy of the events that were inferred on each branch of the cherry in two different ways: *strict event accuracy* and *relaxed event accuracy*. On the one hand, we define the *strict event accuracy* as the ratio of the number of events inferred completely correctly (i.e. with the exact same length and position) over the total number of events generated. On the other hand, we define the *relaxed event accuracy* as the ratio of genes labeled with the correct event over the total number of genes affected by events in the simulated data. In other words, the relaxed ratio focuses on the genes being labeled with the correct event, and not on the number or size of the events. For example, if a deletion of two consecutive genes *a*_1_,*a*_2_ was simulated on a branch by the data generator, and the algorithm inferred two separate deletions *a*_1_ and *a*_2_, the strict event accuracy would be 0%, but the relaxed event accuracy would be 100%.

**Fig. 4:**
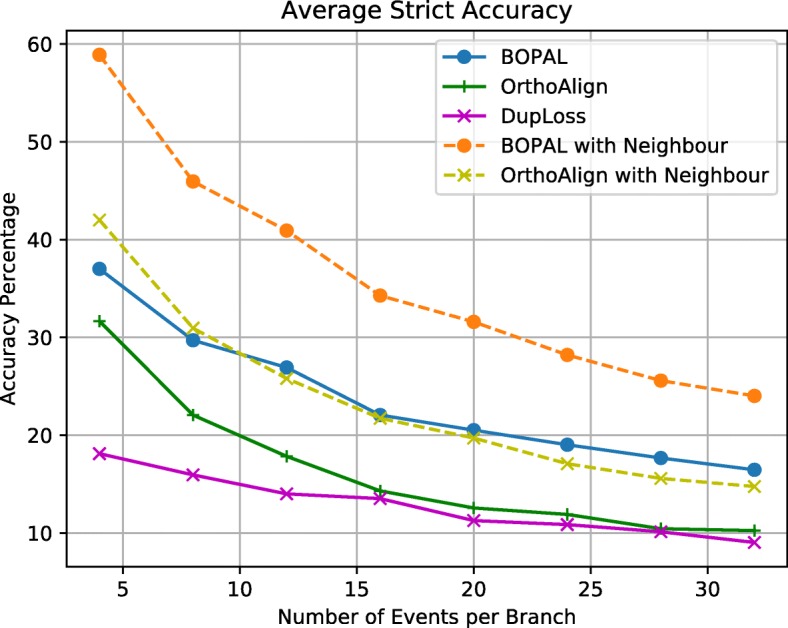
Strict event accuracy

**Fig. 5:**
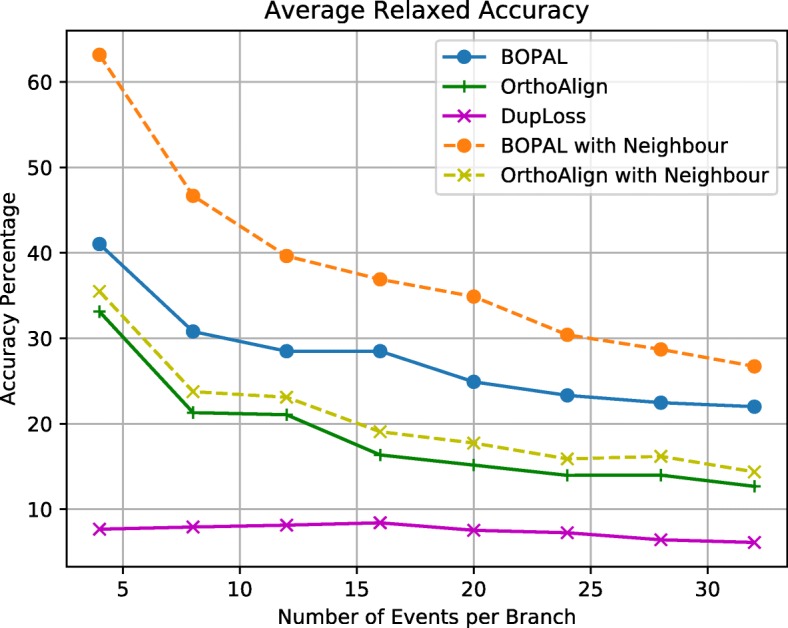
Relaxed event accuracy

The strict and relaxed event accuracy graphs are shown in Figs. 4 and 5. Clearly, inferring accurate events is very difficult in general, and it becomes more difficult as the number of events per branch increases. Note that the tests went up to 32 events per branch, which is much more than what we would typically expect in a real dataset (in the study of 50 *Bacillus* genomes [16], an average of 2.525 events were inferred per branch). In terms of strict event accuracy, BOPAL with a neighboring genome performs the best, with values in the range of 60% to 25%. BOPAL without a neighbor performs similarly to OrthoAlign with a neighbor, while OrthoAlign without a neighbor and DupLoss exhibit the worst performances.

For the relaxed event accuracy, we observe a small improvement of BOPAL both with and without the neighbor compared with the values of strict accuracy. On the other hand, all the other methods (except OrthoAlign without the neighbor) perform worse in terms of relaxed accuracy than for the strict accuracy. To better interpret this result, we analyzed the average size (in number of genes) of all the events inferred completely correctly (the ones that were counted in the strict event accuracy), and found that BOPAL infers more of the longer events on average than its competitors (see Figure S1 of the Supplementary material). BOPAL with a neighbor performs the best all the time, with values ranging between 63% and 27%. Interestingly, it is followed by BOPAL without a neighbor, and then OrthoAlign both with and without a neighbor performing almost similarly. The curve for DupLoss is relatively flat and very low, which is a bit surprising considering that half of the events inferred on each branch are duplications and losses.

**Accuracy on varying genome sizes** We also evaluated how the number of genes in the gene orders affects the accuracy of the different approaches, for a fixed number of events. Basically, we used the same parameters described above, except that *x* was set to 4 (resulting in 16 events per branch plus one inversion), and we used an ancestral genome size *n* varying from 50 to 250. The results, presented in the Supplementary material (Figures S2, S3 and S4), show that all the types of accuracy increase with the number of genes. These results suggest that considering more types of operons in the bacterial genomes could lead to even better inferences of evolutionary scenarios and ancestors.

#### Runtime

**Fig. 6:**
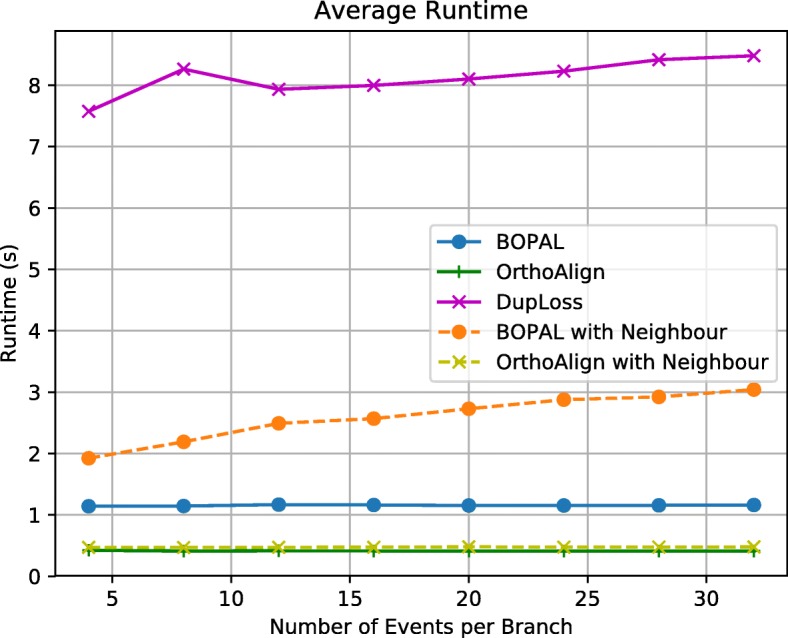
Average runtimes of the different methods compared

We also measured the average runtimes of the 5 different methods (see Fig. 6), using an Intel Core i5 2.5 GHz with 8GB of memory. The runtimes of OrthoAlign and BOPAL without the neighbor are not affected by the number of events. BOPAL with a neighbor is unsurprisingly slower than BOPAL without a neighboring genome, and becomes a little bit slower with more events, which can be explained by the comparisons that have to be made with the neighbor for each rearrangement event to infer it on the correct branch. DupLoss, which uses ILP is unsurprisingly the slowest method of all. BOPAL is a little bit slower in practice than OrthoAlign, with average runtimes of just over 1 s without a neighbor, and between 2 and 3 s with a neighbor, in comparison with average runtimes of approximately 0.5 s for OrthoAlign.

We also measured the speed of our approach on large genomes (values of *n* going up to 1000 genes). BOPAL with a neighbor took a little over 2 min to complete for *n*=1000 (see Figure S5 and Table S1 of the Supplementary material). Even though our methodology is slower than OrthoAlign, it is scalable to large genomes.

### Evaluation on biological datasets

We compared the performance of our algorithm to multiOrthoAlign and DupLoCut on the same biological dataset of 12 *Bacillus* gene orders used in [15] and [17], to which we added the operon annotations (see Table 1 for details on the genomes studied and their operon annotations, and Figure S6 in the Supplementary material for the phylogeny used). BOPAL completed the analysis of the whole tree with a runtime of 6.45 s (on the same Intel Core i5 2.5 GHz with 8GB of memory used for the simulations).

BOPAL inferred 56 duplications, 37 deletions, 8 transpositions and 16 substitutions for a total of 117 events. Based on the results presented in [15] and [17], multiOrthoAlign converged at 123 events and DupLoCut converged to a minimum of 120 events on this dataset (see Table 2 for a summary). However, multiOrthoAlign was restricted to inferring duplications and losses only, just like DupLoCut, whereas BOPAL was using its full evolutionary model. Interestingly, the added constraints of the operon boundaries and the fact that BOPAL does not calculate multiple iterations of the median problem did not result in a scenario with more events. The transposition events inferred by BOPAL probably played a role in the inference of a slightly lower number of events.

**Table 2:** Number of events identified by BOPAL, multiOrthoAlign, DupLoCut on the dataset of 12 *Bacillus* genomes

**Algorithm**	**Reported events**
BOPAL	117
multiOrthoAlign	123
DupLoCut	120

**Fig. 7:**
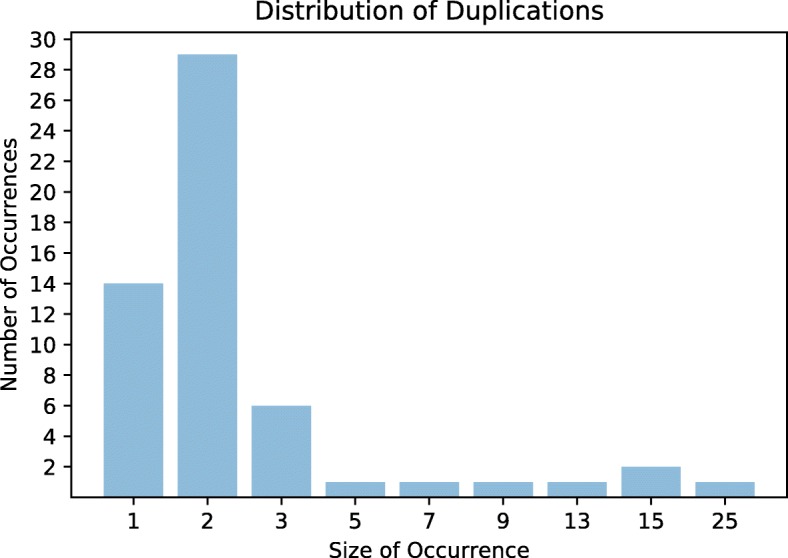
Size distribution of the duplications inferred by BOPAL on the 12 *Bacillus* genomes

**Fig. 8:**
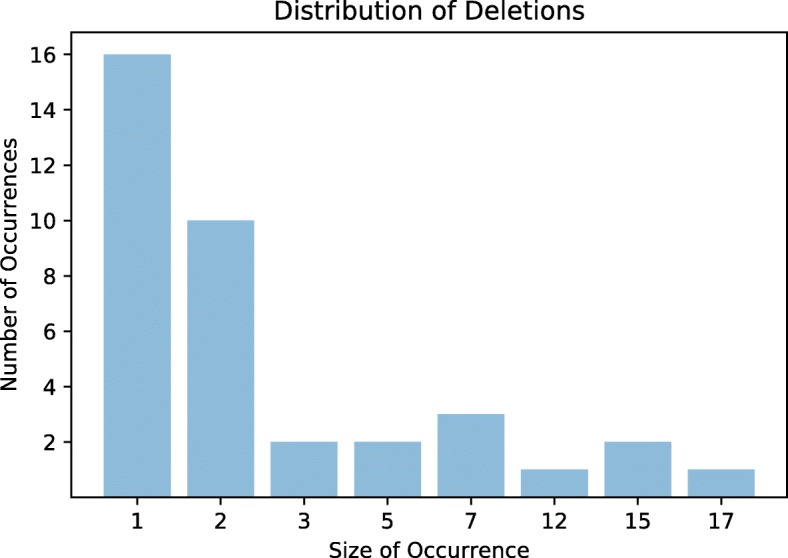
Size distribution of the deletions inferred by BOPAL on the 12 *Bacillus* genomes

87.5% of the duplications inferred by BOPAL were affecting 1, 2 or 3 genes, whereas the rest of the duplications were of size greater than 5, with the largest one being a whole operon duplication of size 25 (see Fig. 7 for the size distribution of duplication events). Similarly, the majority of the inferred deletions were short (see Fig. 8 for the size distribution of deletion events). About 75% of the deletion events were of size 1, 2 or 3, and the rest of them had a length in the range of 5 to 17 genes. Out of the 8 transpositions inferred, three were of size 3, two of size 5, and one of each sizes 6, 12 and 15. Although we were not able to analyze the events inferred by the other methods, it is quite possible that the restrictions of our evolutionary model have given rise to a different but equivalent (in terms of the total number of events) evolutionary history.

## Conclusion

In this paper, we presented BOPAL, a new approach for the inference of realistic evolutionary histories of rRNA and tRNA genes. Our method is based on the identification of orthologous operons, which ultimately helps with the identification of orthologous genes when the genomes have been transformed by many evolutionary events. Our tests on simulated datasets have shown that BOPAL is able to infer more accurate events and ancestors than previous approaches, with a reasonably fast runtime. Results on a biological dataset of 12 *Bacillus* gene orders showed that our method can infer realistic evolutionary scenarios with a similar number of events than existing methods.

Even though the analyses presented here were focused on the evolution of rRNA and tRNA genes, our approach can effectively be adapted to the inference of realistic evolutionary scenarios of any type of genes that are organized into operons. Future work will be devoted to the analysis of more types of operons and other bacterial genera.

In the future, a lot more work will be necessary to improve even more the accuracy of the events inferred. In order to accomplish that, more information will probably be necessary: exact position of the operons and singletons on the genome, intergenic distances between each pair of consecutive genes, and alignments of the flanking regions of each gene considered in the analysis are potential sources of additional information that could be leveraged. Also, similarly to the generalization of OrthoAlign to multiOrthoAlign, it would be interesting to generalize the proposed algorithm to compute the median of three genomes, which could then be used iteratively on a phylogeny with initialized ancestors to further reduce the number of events inferred.

## Supplementary information

**Additional file 1** Supplementary material, evaluation on simulated datasets.

## Data Availability

The software, genome generator and biological dataset are available at: http://bioinformatics.cs.umanitoba.ca/software/BOPAL/.

## Abbreviations

2-SPP: 2-Small phylogeny problem
BOPAL: Bacterial operon aligner
DNA: Deoxyribonucleic acid
DOOR: Database of prokaryotic Operons
FN: False negatives
FP: False positives
Gb: Gigabyte
GHz: Gigahertz
HGT: Horizontal gene transfer
ILP: Integer linear programming
mRNA: Messenger ribonucleic acid
RNA: Ribonucleic acid
rRNA: Ribosomal ribonucleic acid
TP: True positives
tRNA: Transfer ribonucleic acid
