# Supplementary material for: Operon-based approach for the inference of rRNA and tRNA evolutionary histories in bacteria
Source: BMC Genomics. 2020 Apr 16;21(Suppl 2):252. doi: 10.1186/s12864-020-6612-2 (PMC7160887; doi:10.1186/s12864-020-6612-2)
Supplement: Supplementary file 1 — Additional file 1 Supplementary material, evaluation on simulated datasets. [file 12864_2020_6612_MOESM1_ESM.pdf]

Operon-based approach for the inference of rRNA and tRNA evolutionary histories in bacteria

Tomasz Pawliszak, Meghan Chua, Carson K. Leung, and Olivier Tremblay-Savard

Department of Computer Science, University of Manitoba, Canada

Supplementary material

1 Evaluation on simulated datasets

Unless stated otherwise, all the results presented here are averaged over 100 replicates.

1.1 Accuracy on cherries with neighbor

Recall that for this test we used a triplet phylogeny ($L = 3$ leaves), a constant ancestral genome size $n = 120$, $p_{op} = 0.125$ (producing an average operon size of 8.2), $prob_s = 0.35$ (resulting in an average number of singletons and operons of 7.8 and 13.7 respectively), $p_{event} = 0.7$. As for the simulated events, we used one inversion randomly applied to one of the branches of the cherry, and x times a duplication, a deletion, a transposition and a substitution on each branch.

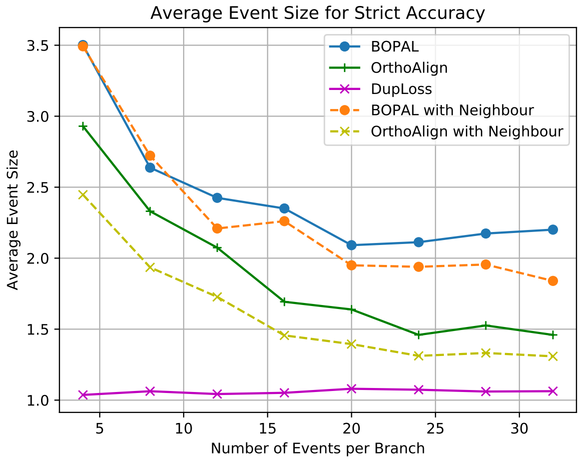

Figure S1: Average size of the events that were inferred completely correctly by the different methods.

Figure S1 presents the average size of the events that were inferred completely correctly (*i.e.* the events considered for calculating the strict accuracy) by the 5 different approaches.

1.1.1 Accuracy on varying genome sizes

For this test, we used the same parameters described above (Section 1.1), except that x was set to 4 (resulting in 16 events per branch plus one inversion), and we used an ancestral genome size n varying from 50 to 250.

Figures S2, S3 and S4 are presenting respectively the F-measure of the reconstructed ancestors, the strict event accuracy and the relaxed event accuracy.

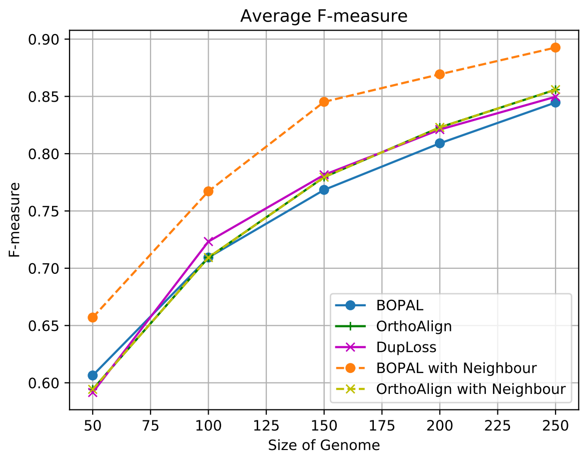

Figure S2: F-measure of the reconstructed ancestral gene orders.

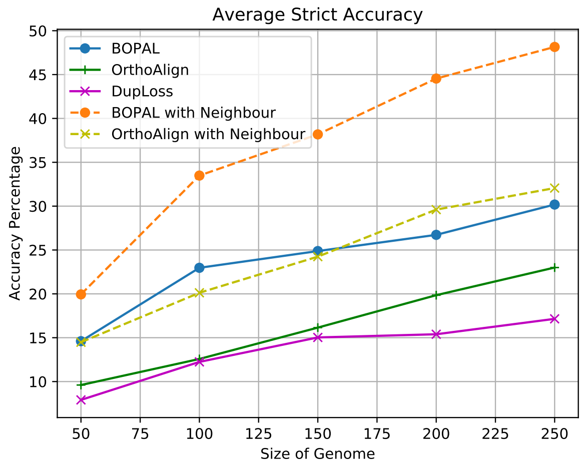

Figure S3: Strict event accuracy.

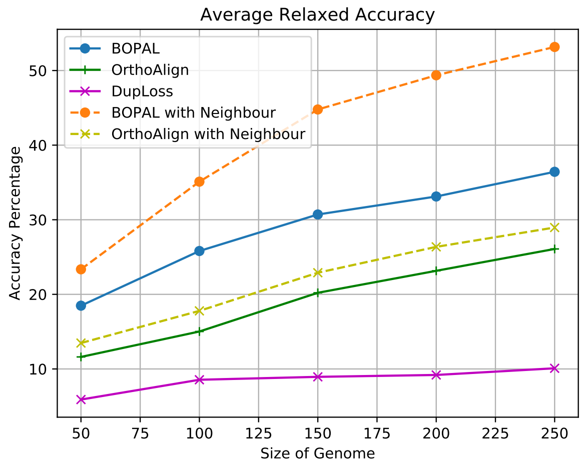

Figure S4: Relaxed event accuracy.

1.2 Runtime on large genomes

To get an evaluation of the scalibility of our method, we measured the runtime of the different approaches on ancestral genome sizes n varying from 200 to 1000 genes (see Figure S5). The other parameters are the same as presented in section 1.1.1. Table S1 shows the average runtimes of the 5 differents approaches for $n = 1000$. Due to the time required to run DupLoss, these results are averaged over only 2 replicates.

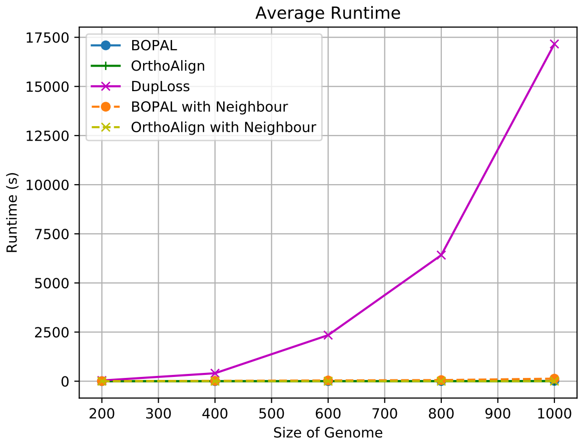

Figure S5: Runtime on large genomes.

Table S1: Average runtimes for $n = 1000$.

Method	Runtime (s)
DupLoss	17161.50
OrthoAlign	1.14
OrthoAlign with neighbour	3.76
BOPAL	14.75
BOPAL with neighbour	130.58

1.3 Evaluation on biological datasets

Figure S6 presents the tree that was used for the analysis of the 12 *Bacillus* genomes. It is the same tree that was used in [1] and [2].

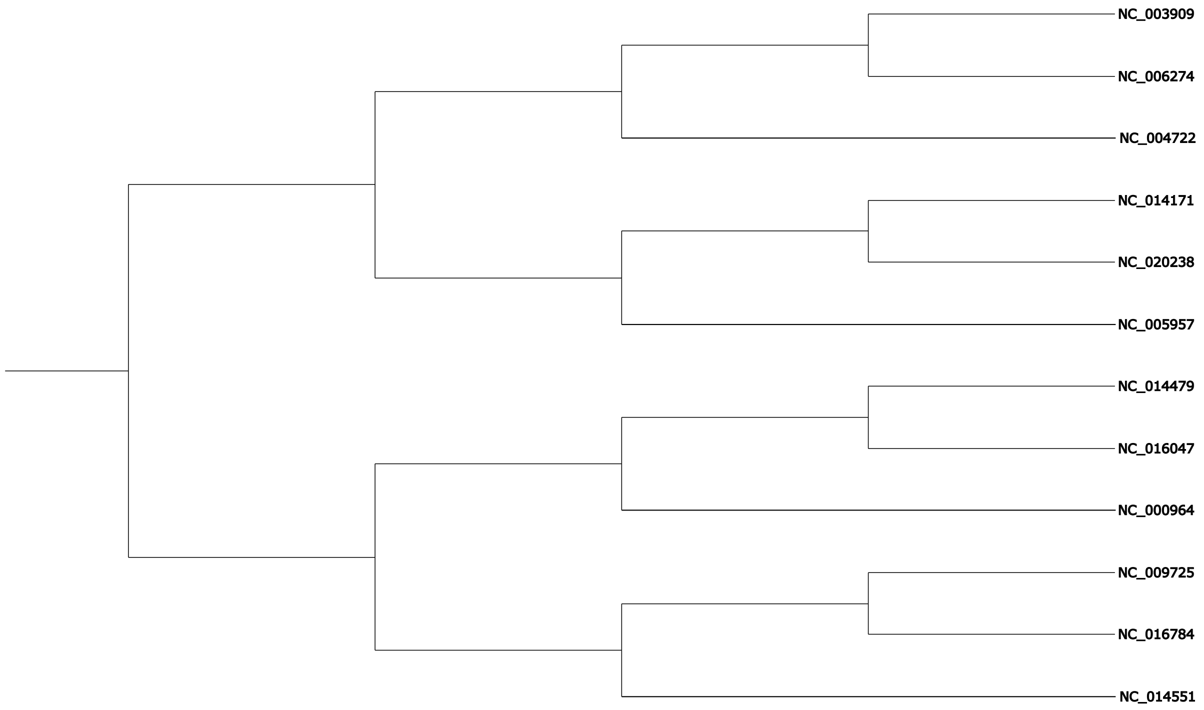

Figure S6: Tree used for the evaluation on biological datasets. The leaf labels represent the Genbank accession numbers of the genomes analyzed.

References

[1] Sandro Andreotti, Knut Reinert, and Stefan Canzar. The duplication-loss small phylogeny problem: from cherries to trees. *Journal of Computational Biology*, 20(9):643–659, 2013.

[2] Billel Benzaid and Nadia El-Mabrouk. Gene order alignment on trees with multiorthoalign. *BMC genomics*, 15(6):S5, 2014.
